# Light-Driven Reversible Shaping of Individual Azopolymeric Micro-Pillars

**DOI:** 10.1038/srep31702

**Published:** 2016-08-17

**Authors:** Federica Pirani, Angelo Angelini, Francesca Frascella, Riccardo Rizzo, Serena Ricciardi, Emiliano Descrovi

**Affiliations:** 1 Department of Applied Science and Technology, Politecnico di Torino, Corso Duca degli Abruzzi 24, Torino IT-10129, Italy; 2 Center for Space Human Robotics@Polito, Istituto Italiano di Tecnologia, C.so Trento 21,10129 Torino, Italy

## Abstract

Azopolymers are known to exhibit a strong light responsivity known as athermal photofluidization. Although the underlying physics is still under debate, athermal photofluidization has been demonstrated to trigger mass-migration according to the polarization of a proper illumination light. Here, a polymer blend is proposed wherein a commercial azo-polyelectrolyte is mixed with a passive polymer. The blend is patterned as an array of micro-pillars that are individually exposed to visible laser illumination. Thanks to the interplay between the two blend components, a reversible and controlled deformation of the micro-pillars by periodically tuning the laser polarization in time is demonstrated. A reduced mobility of the azo-compound allows to repeatibly elongate and rotate micro-pillars along specific directions, with no significant material flow outisde the initial volume and no significant degradation of the structure morphology over several cycles. The proposed work suggests new degrees of freedom in controlling the mechanical features of micro-patterned light-responsive materials that can be usefully exploited in many application fields.

In the last two decades, micro and nano-structured polymeric films have attracted significant interest because of their promising potential application in many areas, including micro-fluidics, smart surfaces, photonics, tissue engineering and the like. Nowadays, a plethora of processing technologies are available for fabricating complex polymeric architectures which are mostly static in nature, i.e. they cannot be morphologically modified once fabricated. However, light-responsive materials such as azobenzene polymeric compounds (generally referred to as azopolymers) can overcome such a limitation, as they exhibit a strong light-induced mechanical effects occurring either at the microscopic^1^,^2^,^3^,^4^ or macroscopic scale^5^,^6^,^7^. In azopolymeric films, a directional mass-migration effect can be triggered depending on the radiation wavelength, intensity, polarization state and topological charge, e.g. in vortex beams^8^,^9^,^10^,^11^. In some cases, such an effect is termed athermal photofluidization, thus indicating a photo-softening and a corresponding directional deformation of the azopolymer due to the cyclic isomerization of the azobenzene^12^. Alternatively, the isomerization process has been invoked to explain a re-orientation of the polymer backbones, which may result in viscoplastic mechanical stresses anisotropically deforming the material^13^,^14^. Despite the underlying mechanism is still unclear to some extent^14^,^15^,^16^, mass-migration in azopolymers has been widely exploited in the past for fabricating large-area periodic microstructures also known as Surface Relief Gratings (SRG) by exploiting intensity and/or polarization interference^17^,^18^. Recently, azopolymers have been proposed for cell-growth applications^19^,^20^,^21^,^22^,^23^, wherein the possibility of multiple writing/erasure of SRG and related morphology can be advantageously exploited to reconfigure the cellular substrate^24^. In such a situation, lithographed azopolymeric patterns that could be subsequently modified by irradiation in controlled conditions are particularly attractive^25^,^26^,^27^. For instance, irradiation with a linearly polarized light can elongate circular micro-pillars resulting in an ellipsoidal shape, wherein the elongation is along the polarization direction of the illumination beam^28^. However, this process is hardly reversible on isolated azopolymeric micro-objects, as an excessive photofluidization results in a material flow outside the initial volume^29^.

In this work, we propose a light-responsive polymeric micro-pattern that can be arbitrarily and reversibly modified upon proper illumination conditions. More specifically, a mixture made of an azopolymer (PAZO)^30^ and a synthetic resin (PMMA) is transferred as a squared lattice of circular pillars over large area, by means of a soft lithographic technique. Recently, PMMA has been demonstrated to induce a symmetry-breaking effect in the photo-deformation of core-compartimentalized PAZO within Janus nanoparticles^31^. On a larger scale, we show here that a small fraction of PMMA within a PAZO-PMMA mixture can improve the mechanical response to light-stimuli, allowing the deformation of micro-pillars to be reversibly controlled in direction over several cycles.

## Results and Discussion

**Figure 1:**
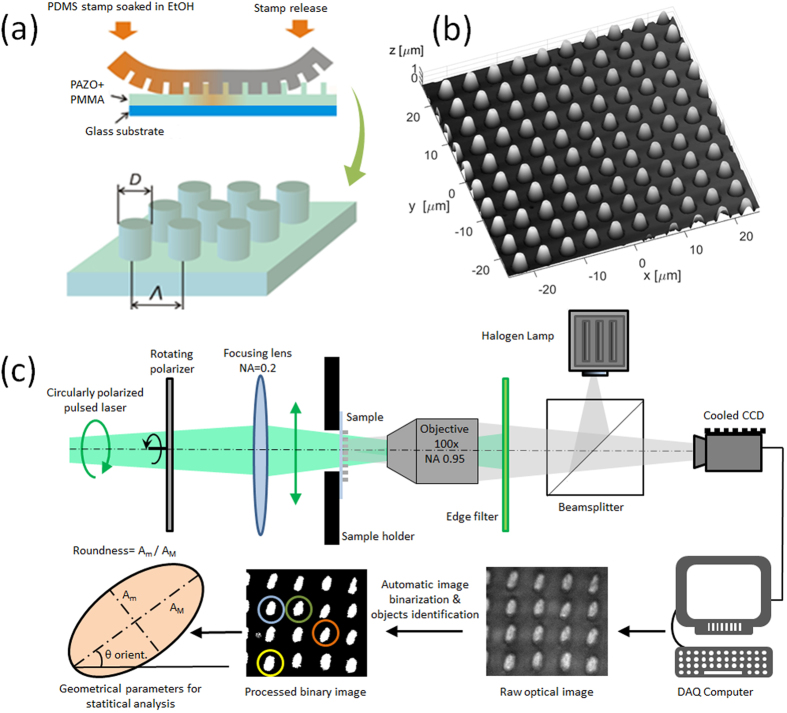
Sample and experimental setup. (**a**) Sketch of the soft lithography method for micro-pillar fabrication on glass substrate (pillar diameter D = 3 μm, period Λ = 5 μm, height h = 1.5 μm); (**b**) 3D representation of an AFM topography map of the micro-pillar array; (**c**) sketch of the optical setup, the data acquisition and the processing flow.

A mixture of commercially available poly[1-[4-(3-carboxy-4-hydroxyphenyl-azo)benzene sulfonamido]-1,2-ethanediyl, sodium salt] (PAZO) and (poly(methyl methacrylate) (PMMA), (both from Sigma-Aldrich) is prepared with ratio 10:1, as detailed in the Experimental Section. In Fig. 1a, a sketch for the soft-lithographic method used for PAZO-PMMA patterning is illustrated. As a result, an array of well-defined azopolymeric micro-pillars is then obtained. Scanning Electron Microscopy and Atomic Force Microscopy analysis (see Fig. 1b) reveal circular pillars with a D = 3 μm diameter and h = 1.5 μm height, arranged as a squared lattice with Λ = 5 μm periodicity.

The setup for triggering and monitoring the micro-pillar modification is shown in Fig. 1c. A polarization-controlled pulsed laser beam (10 ps pulse duration, wavelength λ = 490 nm) is used to irradiate the sample. The micro-pillar surface is monitored by means of a wide-field white-light microscope in reflection mode. Upon optical image collection and analysis, some geometrical parameters, including the major axis AM, the minor axis Am and the orientation θ of the pillars, where θ is the angle between the major axis and a horizontal axis, are extracted for all individual objects within each image. In addition, mean values are calculated in order to add significance and robustness to the overall description of the collective shape modification involving a plurality of pillars.

**Figure 2:**
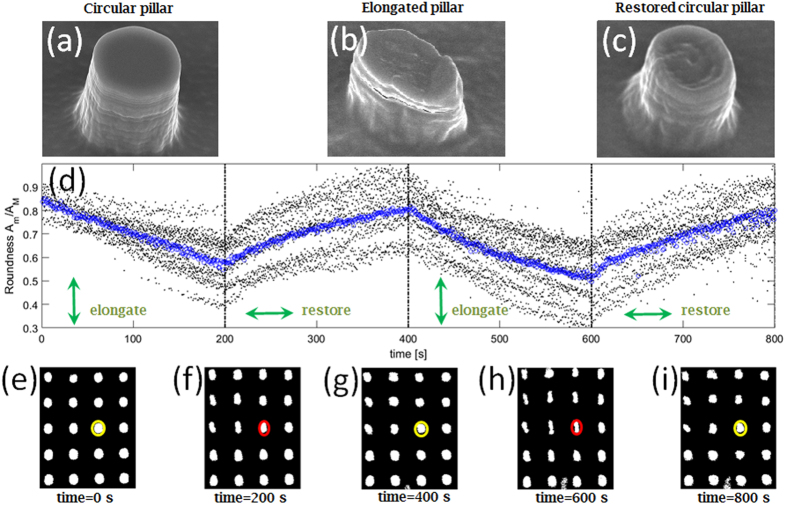
Pillar reversible elongation. Exemplary SEM pictures of individual pillars: (**a**) before laser irradiation (circular cross section), (**b**) after a single laser exposure with linear polarization (elongated, elliptical cross section), (**c**) after two laser exposures with two orthogonal polarizations (restored circular cross section); (**d**) time-resolved roundness values for pillars during laser irradiation with time-varying polarization states (black dots: roundness of individual pillars, blue circles: mean value over pillars in each frame); (**e–i**) binarized optical images of micro-pillars at relevant times during pillar light-modification. An accelerated (30x) live sequence of the two-cycle elongation-restoration process depicted here is provided in movies S1 (raw frames) and S2 (binarized frames) in the Supporting Information.

As-fabricated micro-pillars possess a well-defined circular cross section (Fig. 2a) that becomes strongly elongated upon illumination by a linearly polarized laser beam (Fig. 2b). The direction of the elongation is parallel to the polarization of the incident beam, in accordance to previous studies on photofluidization^25^,^26^,^27^,^28^. Further shape modifications, including feature erasure, can be performed by varying the polarization state of the incident laser beam^26^. As an example, it has been suggested that a linearly polarized field impinging on an isolated slab of azopolymer can trigger a diffusive effect such that the slab gets stretched along the polarization direction and simultaneously shrunk along the orthogonal direction^14^. Here, pillars are re-irradiated with a linear polarization, which is rotated by 90 degrees with respect to a previous irradiation. A typical result is shown in Fig. 2c. Despite some detrimental effects on the morphology quality, a substantially circular cross section is restored by triggering a laser-induced stretching along the minor axis of the elongated pillars. A similar result has been recently found on the light-tuning of the pore shape in azopolymeric breath figures^27^. The reversible elongation of the pillars can be iterated several times, with a slow progressive degradation of the structures. For a quantitative analysis, in Fig. 2d a mean roundness parameter is plotted against time, as a polarization-variable laser illumination is performed. The mean roundness is evaluated time by time as the average of the ratio Am(t)/AM(t) over 20 micro-pillars included in the optical images that are collected, binarized and analyzed as described above. In Fig. 2e–i, exemplary binarized images are shown corresponding to relevant times during the light-induced modification of the pillars. More specifically, Fig. 2e is related to the initial pillar state (such as in Fig. 2a), Fig. 2f,h are related to an elongated state (such as in Fig. 2b) and Fig. 2g,i are related to a restored circular state (such as in Fig. 2c). In Fig. 2d we observe that elongation-restoration cycles can be repeated upon irradiation with the very same laser power, by simply rotating the incident polarization by 90 degrees, from a vertical to a horizontal orientation and vice-versa. In time cycles lasting about 400 s, almost circular pillars (mean roundness about 0.9, as estimated from Fig. 2e) are elongated along the vertical direction (mean roundness down to 0.6, as estimated from Fig. 2f,h) and then restored back to a circular shape (Fig. 2g,i). This reversible pillar modification is advantageously promoted by an improved stiffness of the polymeric mixture due to the presence of a stabilizing PMMA component, as suggested elsewhere in other co-polymer matrices containing azobenzenes^5^. In this way, the mass migration triggered by photofluidization is prevented to flow outside the pillar volume even without external constraints^29^.

**Figure 3:**
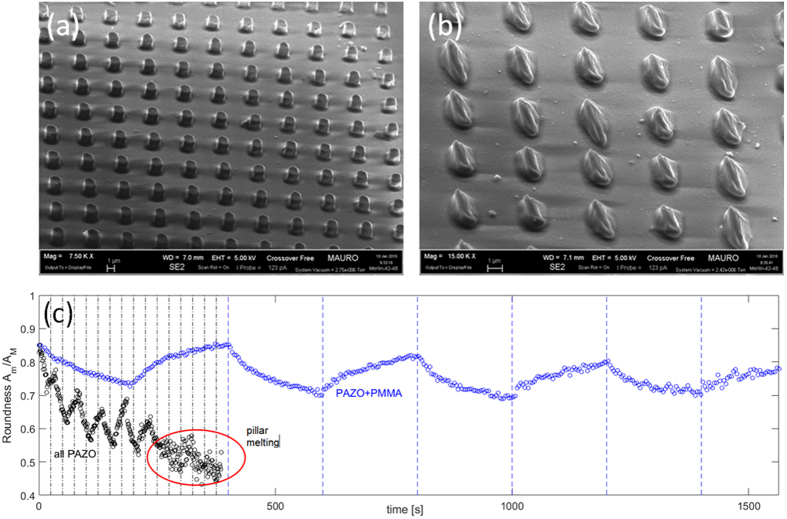
Stability of pillar deformation cycles. Exemplary SEM pictures of individual all-PAZO pillars: (**a**) before and (**b**) after a laser exposure with linear polarization (irradiation time 400 s). (**c**) Time-resolved mean roundness values for pillars during laser irradiation with time-varying polarization states (black circles: PAZO pillars, blue circles: PAZO+PMMA pillars).

Worth to note here that illuminated PAZO-only micro-pillars are prone to quickly lose their regular shape because of the strong mass diffusion away from the volume initially occupied in their as-fabricated state. For comparison purposes, a SEM picture of all-PAZO pillars before and after an overall irradiation time of 400 s is shown in Fig. 3a,b. The softening of PAZO pillars due to the athermal photofluidization hampers the full recovery of a regular pillar shape because of the progressive leakage of polymer out of the initial confined volume. The elongation-restoration mechanism can be hardly repeatable over many cycles, as demonstrated by the time-dependent mean roundness plot shown in Fig. 3c. In fact, when several elongation-restoration steps are performed in sequence, the PAZO pillars get completely melted and the pillar cross section cannot be optically recognized anymore.

**Figure 4:**
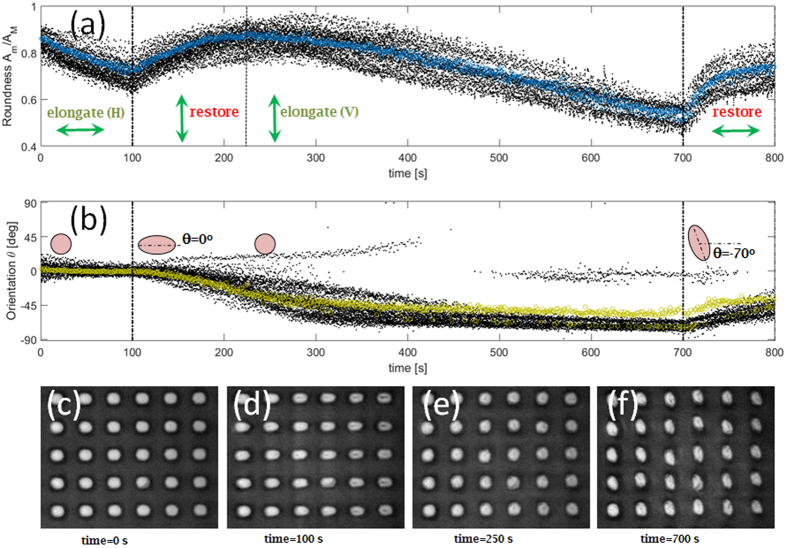
Pillar flipping. (**a**) Time-resolved roundness values (black dots: roundness of individual pillars, blue circles: mean value per frame) and (**b**) time-resolved orientations (black dots: orientation of individual pillars, green squares: mean value per frame) for pillars during laser irradiation with time-varying polarization states; (**c–f**) optical images of micro-pillars at relevant times during pillar light-modification. An accelerated (30x) live sequence of the horizontal/vertical elongation process depicted here is provided in movie S3 in the Supporting Information.

The stretching/shrinking effect described above can be extended for longer time periods. In such a case, a new deformation of the micro-pillars can occur, eventually along a different orientation as determined by the illumination polarization. Figure 4a,b illustrates the time-evolution of the mean roundness and the mean orientation angle θ(t) calculated over 30 micro-pillars exposed to a laser beam sequentially switching between two orthogonal linear polarizations. During the first 100 s, as-fabricated circular micro-pillars (Fig. 4c) are illuminated with an H-polarized beam such that a horizontal elongation is produced, corresponding to a mean roundness of about 0.7 (estimated from Fig. 4d). When the polarization is rotated by 90 degrees (V-polarization), the initial circular shape is restored (mean roundness 0.9) in 130 s irradiation (estimated from Fig. 4e). However, if the V-polarized illumination is kept for additional time, the mean roundness parameter starts to decrease again, the elongation occurring along a different direction. In fact, from 100 s to 400 s, the mean orientation angle θ experiences a rather quick change from 0 degrees (horizontally oriented micro-pillars) to nearly −70 degrees (almost vertically oriented micro-pillars). After 700 s, the micro-pillars are well elongated (roundness 0.5) and flipped as shown in Fig. 4f. Afterwards, upon a further switch to H-polarization, a restoring force is triggered, such that the roundness parameter starts to increase again. Worth to note here that the maximum orientation change we could induce on already elongated pillars with a given orientation has been generally limited to angles smaller than 90 degrees only. In the specific case illustrated in Fig. 4, after the first horizontal elongation, pillars are hampered to fully orient in a vertical direction, despite a vertically oriented polarization has been used for illumination. As an explanation, we speculate that some degree of polarization-induced irreversible plasticity within the PAZO-PMMA matrix might occur during the very first exposure^14^. In alternative, a phase separation between PMMA and PAZO due to the light-induced diffusion might constrain the mass-migration mobility^31^.

**Figure 5:**
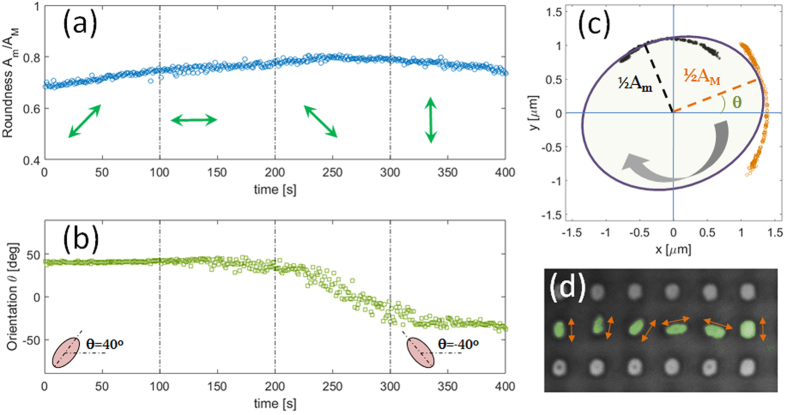
Pillar rotation. (**a**) Time-resolved almost constant roundness and (**b**) time-resolved orientation (mean value per frame) for pillars during laser irradiation with time-varying polarization states; (**c**) rotational trajectory traced by the minor (black asterisks) and the major (orange circles) axis of an equivalent “average ellipse” (blue lineprofile) during clockwise rotation; (**d**) optical image of individually modified pillars (in green) with varying orientation as indicated by the orange arrows. An accelerated (30x) live sequence of the rotation process depicted in (**a–c**) is provided in movie S4 in the Supporting Information.

By rotating the polarization of the incident laser by angular steps smaller than 90 degrees, it is possible to induce micro-pillars to smoothly orient accordingly, while still keeping a given elongation. For example, in Fig. 5a, elongated pillars are observed to maintain a roughly constant (mean) roundness value during a 400 s illumination wherein the polarization is rotated by 45 degrees each 100 s (green arrows in the figure). In this case, light-induced deformations are provided such that an overall rotation of the elongated pillars directly occurs without an intermediate restore of the initial circular shape. As shown in Fig. 5b, the mean orientation angle suggests that pillars are substantially experiencing a smooth rotation of about 80 degrees according to the instantaneous polarization direction (with some delay due to the viscosity of the polymer). In order to better illustrate this effect, starting from the estimated mean values for A_m_(t), A_M_(t) and θ(t) in each time frame, the trajectories defined by the ray vectors associated to the pillar minor axis and major axis are traced on an xy-plane that ideally contains the pillar top surface (black circles for the minor axis and orange circles for the major axis in Fig. 5c). As a result, a ‘mean’ elliptical pillar can be drawn having a minor axis and a major axis oriented according to the instantaneous value of θ(t). Such a graphical representation of the ‘mean’ elliptical pillar in time provides an intuitive description of the substantially rigid rotation experienced during the polarization changes. A movie of the full rotation occurring in the 400 s time interval is shown as video S5 in Supporting Information.

The fine control on the elongation and orientation of PAZO-PMMA structures demonstrated above can also allow a tuning of the surface pattern on an individual-pillar basis. In fact, if illumination is provided as a localized light spot (e.g. by means of a a focusing system), micro-pillars can be sequentially modified according to the desired polarization state. In Fig. 5d, a SEM image of several pillars sequentially elongated in different directions is illustrated.

## Conclusion

In conclusion, we demonstrated that the mixture of an azopolymer with a passive component such as PMMA can improve the mechanical characteristics of individual micro-objects suitable for deformation using light. As an exemplary micro-structure, we considered micrometer-sized pillars that can be reversibly elongated and rotated with a rather good degree of control, upon optimal choice of the radiation wavelength and polarization. While micro-pillars have been chosen in order to allow a quick and statistically meaningful optical characterization, an analogous mechanical light-responsivity is expected to occur also in nano-sized objects. The findings here presented are promising in useful applications such as tunable smart architectures including, e.g. photonic crystals and optical metamaterials^32^,^33^, and dynamically wettable surfaces^34^,^35^,^36^.

## Experimental Methods

### Sample Fabrication

A mixture of commercially available poly[1-[4-(3-carboxy-4-hydroxyphenyl-azo)benzene sulfonamido]-1,2-ethanediyl, sodium salt] (PAZO) and (poly(methyl methacrylate) (PMMA), (both from Sigma-Aldrich) is prepared as follows. A solution of PMMA with molecular weight (Mw) of ~15000 g/mol. is dissolved in N,N-Dimethylformamide at a concentration of 4 wt%. PAZO dissolved in methanol (concentration 25 mg/mL) is added to the PMMA solution, at 10:1 ratio and then mechanically stirred and sonicated for few minutes until a homogeneous blend is formed. An amount of 20 μl of PAZO-PMMA is casted onto a glass substrate, previously washed in acetone, rinsed with isopropanol and dried with a nitrogen flow.

An elastomeric stamp, fabricated using conventional soft lithography molding techiniques, is used to pattern a centimetric area of micro-pillar array. The PDMS stamp is prepared by mixing Sylgard 184 Dow Corning elastomer with the curing agent at a 10:1 ratio. The mixture is then casted onto a silicon master whose surface has been patterned with the complementary structures, subsequently degassed in a dessicator and left to cure at 60 °C for 2 hours. The prepared PDMS stamp is soaked in ethanol for few minutes and then gently pressed on the PAZO-PMMA film on glass.

The sample is placed in a vacuum dessicator for 2 hours in order to enhance patterning and let the polymer blend to fill in the PDMS template. As a next step, the sample is dried in oven, until the solvent is completely evaporated (3 hours at 60 °C) before the final removal of the PDMS stamp. An array of well-defined azo-polymeric micro-pillars over a large area is then obtained.

### Optical Conditioning and Monitoring

A slightly divergent pulsed laser beam (10 ps pulse duration, wavelength λ = 490 nm) with an initial circular polarization is polarization-filtered through a polarizer. As the polarizer can be rotated about the optical axis, the resulting beam can be linearly polarized along any arbitrarily controlled polarization direction. A low Numerical Aperture (NA = 0.2) lens focuses the laser beam onto the back of the fabricated sample. A laser power of 1.3 mW covers a homogeneous circular illumination area with radius about 25 μm. The micro-pillar surface is monitored by means of a wide-field white-light microscope in reflection mode, employing a 100x, NA = 0.95 objective (Olympus). A computer-controlled 24 bit CCD (Apogee Ascent) records the white light images after proper laser-filtering (Semrock RazoEdge MaxLine 532). The CCD is operated in such a way that optical images are collected at a frame rate of 1 Hz. An on line image processing code takes the raw data from the CCD, performs a proper thresholding, then pixel binarization and pattern recognition operations of unconnected areas. As a result, a set of generally elliptical individual objects is recognized from each image. Some geometrical parameters, including the major axis A_M_, the minor axis A_m_ and the orientation θ of the ellipses, where θ is the angle between the major axis and a horizontal axis, are extracted for all individual objects in each image and made available for further analysis. In addition, mean values are calculated in order to add significance and robustness to the overall description of the collective shape modification involving a plurality of pillars.

## Additional Information

**How to cite this article**: Pirani, F. *et al*. Light-Driven Reversible Shaping of Individual Azopolymeric Micro-Pillars. *Sci. Rep.* **6**, 31702; doi: 10.1038/srep31702 (2016).

## Supplementary Material

Supplementary Information

Supplementary Video S1

Supplementary Video S2

Supplementary Video S3

Supplementary Video S4

Supplementary Video S5

## References

[b1] Mahimwalla Z. S. . Azobenzene Photomechanics: Prospects and Potential Applications. Polym. Bull. 69, 967–1006 (2012).

[b2] Gritsai Y., Goldenberg L. M., Kulikovska O. & Stumpe J. 3D Structures Using Surface Relief Gratings of Azobenzene Materials. J. Opt. A. Pure Appl. Opt. 10, 125304 (2008). 10.1364/ol.33.001309 18552941

[b3] Ishitobi H. . Nanomovement of Azo Polymers Induced by Longitudinal Fields. ACS Photonics 1, 190–197 (2014).

[b4] Sobolewska A., Bartkiewicz S. & Priimagi A. High-Modulation-Depth Surface Relief Gratings Using s–s Polarization Configuration in Supramolecular Polymer–Azobenzene Complexes. J. Phys. Chem. C. 118, 23279–23284 (2014).

[b5] Ube T., Takado K. & Ikeda T. Photomobile Materials with Interpenetrating Polymer Networks Composed of Liquid-Crystalline and Amorphous Polymers. J. Mater. Chem. C. 3, 8006–8009 (2015).

[b6] Zeng H. . Light-Fueled Microscopic Walkers. Adv. Mater. 27, 3883–3887 (2015). 26033690 10.1002/adma.201501446 PMC4660875

[b7] Takashima Y. . Expansion-Contraction of Photoresponsive Artificial Muscle Regulated by Host-Guest Interactions. Nat. Commun. 3, 1270 (2012). 23232400 10.1038/ncomms2280 PMC3535346

[b8] Priimagi A. & Shevchenko A. Azopolymers-Based Micro- and Nanopatterning for Photonic Applications. J. Polym. Sci. Part B:Polym. Phys. 52, 163–182 (2014).

[b9] Juan M. L. . Multiscale Model for Photoinduced-Molecular Motion in Azo Polymers. ACS Nano 3, 1573–1579 (2009). 19438196 10.1021/nn900262e

[b10] Ambrosio A., Marrucci L., Borbone F., Roviello A. & Maddalena P. Light-Induced Spiral Mass Transport in Azo-Polymer Films Under Vortex-Beam illumination. Nat. Commun. 3, 989 (2012). 22871808 10.1038/ncomms1996 PMC3432464

[b11] Accary J. B. & Teboul V. How Does the Isomerization Rate Affect the Photoisomerization-Induced Transport Properties of a Doped Molecular Glass-Former? J. Chem. Phys. 139, 034501 (2013). 23883041 10.1063/1.4813410

[b12] Hurduc N. . Direct Observation of Athermal Photofluidization in Azo-Polymer Films. Soft Matter 10, 4640–4647 (2014). 24833017 10.1039/c4sm00397g

[b13] Yadavalli N. S., Korolkov D., Moulin J. F., Krutyeva M. & Santer S. Probing Opto-Mechanical Stresses Within Azobenzene-Containing Photosensitive Polymer Films by a Thin Metal Film Placed Above. ACS Appl. Mater. Interfaces 6, 11333–11340 (2014). 24995460 10.1021/am501870t

[b14] Yadavalli N. S. . Comparative Study of Photoinduced Deformation in Azobenzene Containing Polymer Films. Soft Matter 12, 2593–2603 (2016). 26853516 10.1039/c6sm00029k

[b15] Galinski H. . Instability-induced pattern formation of photoactivated functional polymers. PNAS 111, 17017–17022 (2014). 25404346 10.1073/pnas.1409718111 PMC4260583

[b16] Bin J. & Oates W. S. A Unified Material Description for Light Induced Deformation in Azobenzene Polymers. Sci. Rep. 5, 14654 (2016). 26437598 10.1038/srep14654 PMC4594130

[b17] Yadavalli N. S., Saphiannikova M. & Santer S. Photosensitive Response of Azobenzene Containing Films Towards Pure Intensity or Polarization Interference Patterns. Appl. Phys. Lett. 105, 051601 (2014).

[b18] Kang H. S., Lee S. & Park J.-K. Monolithic, Hierarchical Surface Reliefs by Holographic Photofluidization of Azopolymer Arrays: Direct Visualization of Polymeric Flows Adv. Funct. Mater. 21, 4412–4422 (2011).

[b19] Baac H. . Submicron-Scale Topographical Control of Cell Growth Using Holographic Surface Relief Grating. Materials Science and Engineering C 24, 209–212 (2004).

[b20] Rocha L. . Azobenzene Based Polymers as Photoactive Supports and Micellar Structures for Applications in Biology. J. Photochem. Photobiol. A: Chemistry 291, 16–25 (2014).

[b21] Hurduc N. . Azo-Polysiloxanes as New Supports for Cell Cultures. Materials Science and Engineering C 33, 2440–2245 (2013). 23498280 10.1016/j.msec.2013.01.012

[b22] Rianna C., Ventre M., Cavalli S., Radmacher M. & Netti P. A. Micropatterned Azopolymer Surfaces Modulated Cell Mechanics and Cytoskeleton Structure. ACS Appl. Mater. Interfaces 7, 21503–21510 (2015). 26372777 10.1021/acsami.5b06693

[b23] Barillé R., Janik R., Kucharski S., Eyer J. & Letournel F. Photo-Responsive Polymer with Erasable and Reconfigurable Micro- and Nano- Patterns: an *In Vitro* Study for Neuron Guidance. Colloids and Surfaces B: Biointerfaces 88, 63–71 (2011). 21764267 10.1016/j.colsurfb.2011.06.005

[b24] Rianna C. . Reversible Holografic Patterns on Azopolymers for Guiding Cell Adhesion and Orientation. ACS Appl. Mater. Interfaces 7, 16984–16991 (2015). 25876082 10.1021/acsami.5b02080

[b25] Lee S., Kang H. S. & Park J. K. High-Resolution Patterning of Various Large-Area, Highly Ordered Structural Motifs by Directional Photofluidization Lithography: Sub-30-nm Line, Ellipsoid, Rectangle, and Circle Arrays. Adv. Funct. Mater. 21, 1770–1778 (2011).

[b26] Lee S., Kang H. S. & Park J. K. Directional Photofluidization Lithography: Micro/Nanostructural Evolution by Photofluidic Motions of Azobenzene Materials. Adv. Mater. 24, 2069–2103 (2012). 22454301 10.1002/adma.201104826

[b27] Wang W. . Directional Photomanipulation of Breath Figure Arrays. Angew. Chem. 126, 12312–12315 (2014). 10.1002/anie.201407230 25243818

[b28] Kang H. S. . Light- Induced Surface Patterning of Silica. ACS Nano 9, 9837–9848 (2015). 26389813 10.1021/acsnano.5b03946

[b29] Lee S., Kang H. S., Ambrosio A., Park J. K. & Marrucci L. Directional Superficial Photofluidization for Deterministic Shaping of Complex 3D Architectures. ACS Appl. Mater. Interfaces 7, 8209–8217 (2015). 25816857 10.1021/acsami.5b01108

[b30] Frascella F., Angelini A., Ricciardi S., Pirri F. & Descrovi E. Surface-Relief Formation in Azo-Polyelectrolyte Layers with a Protective Polymer Coating. Opt. Mat. Express 6, 444–450 (2016).

[b31] Zhou X., Du Y. & Wang X. Azo Polymer Janus Particles and Their Photoinduced, Symmetry-Breaking Deformation. ACS Macro Letters 5, 234–237 (2016). 10.1021/acsmacrolett.5b00932 35614684

[b32] Hong J.-C., Park J.-H., Chun C. & Kim D.-Y. Photoinduced Tuning of Optical Stop Bands in Azopolymer Based Inverse Opal Photonic Crystals. Adv. Funct. Mater. 17, 2462–2469 (2007).

[b33] Arbabi A., Horie Y., Bagheri M. & Faraon A. Dielectric Metasurfaces for Complete Control of Phase and Polarization with Subwavelength Spatial Resolution and High Transmission. Nat. Nanotechnol. 10, 937–943 (2015). 26322944 10.1038/nnano.2015.186

[b34] Zillohu A. U. . Biomimetic Transferable Surface for a Real Time Control over Wettability and Photoerasable Writing with Water Drop Lens, Sci. Rep. 4, 7407 (2014). 25491016 10.1038/srep07407 PMC4261171

[b35] Wagner N. & Theato P. Light-induced wettability changes on polymer surfaces. Polymer 55, 3436–3453 (2014).

[b36] Shin S. . Bio-Inspired Extreme Wetting Surfaces for Biomedical Applications, Materials 9, 116 (2016). 10.3390/ma9020116 PMC5456462 28787916

