# Supplementary material for: Light-Driven Reversible Shaping of Individual Azopolymeric Micro-Pillars
Source: Sci Rep. 2016 Aug 17;6:31702. doi: 10.1038/srep31702 (PMC4987756; doi:10.1038/srep31702)
Supplement: Supplementary Information [file srep31702-s1.doc]

**Supplementary Information for**

Light-Driven Reversible Shaping of Individual Azopolymeric Micro-Pillars

Federica Pirani, Angelo Angelini, Francesca Frascella, Riccardo Rizzo, Serena Ricciardi, and Emiliano Descrovi

**Supplementary Videos**

**Supplementary Video S1.** Accelerated (30x) live sequence of the two-cycle elongation-restoration process depicted in Figure 2 (raw data)

**Supplementary Video S2.** Accelerated (30x) live sequence of the two-cycle elongation-restoration process depicted in Figure 2 (binarized data)

**Supplementary Video S3.** Accelerated (30x) live sequence of the horizontal/vertical elongation process depicted in Figure 4.

**Supplementary Video S4.** Accelerated (30x) live sequence of the pillar rotation process depicted in Figure 5.

**Supplementary Video S5.** Full rotation of a "mean pillar" sketched according to the trajectories followed by the minor and the major axis as experimentally collected in time.
